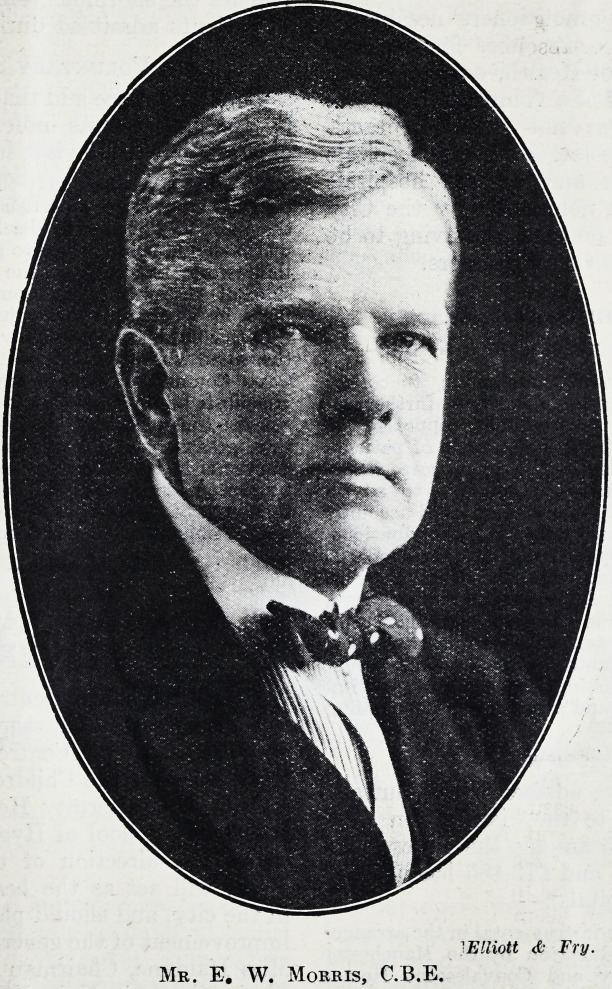# Hospital Men of Mark: Mr. E. W. Morris, C.B.E.

**Published:** 1924-01

**Authors:** 


### HOSPITAL MEN OF MARK.

### MR. E. W. MORRIS, C.B.E., of "The London."

THE fame of the London Hospital is, as Mr. Morris himself says in his history of the institution, a thing of comparatively recent growth. Yet for many generations the Hospital has formed part of the very life of the East-Ender. "These people do not simply notice the Hospital; they love it. Hardly a house but at some time or other has lent a patient to the Hospital to be coaxed back to health and happiness; not a family but has had father or mother, brother or sister or child lying within its walls." It is from very small beginnings that this great institution has sprung. It is the result of "a little meeting of seven men which took place in the bar-parlour of the Feathers Tavern, Cheapside, in the evening of September 23, 1740, when 'a motion was made whether with the sum already subscribed (100 guineas) it would be proper to begin the said Charity.'" Mr. Morris asks "Why after 500 years of apparent neglect, this sudden awakening to the needs of the sick poor?" It would appear that the health of London was never worse than at that period; epidemics of typhus and smallpox arrived periodically, drunkenness was exceedingly prevalent, and the bad atmosphere, partly the result of the abominable window-tax, helped all diseases to flourish. Therefore the foundation of the Hospital was, even if long delayed, particularly timely.

The real founder of the London Hospital was Mr. John Harrison, surgeon, whose bold and progressive policy was of incalculable value in those early days. "His zeal indeed aroused much jealousy and a charge was brought against him of dishonesty, from which however he was entirely acquitted. He was a very popular teacher and constantly introduced pupils to study at the Hospital." The foundation of the Hospital as a great teaching school was, however, the work of Sir William Blizard, whose connection with it was said to be the pride of his life. He was the friend of John Howard, who three times visited the Hospital, suggesting "many useful observations with regard to rendering such asylum of the afflicted sweet and salutary." Howard condemned the raising of dust in the wards, advocated cross-ventilation, suggested that bedding should be laid in the sun as often as possible and flock from mattresses washed and then baked. Blizard himself was keenly aware of the need of cleanliness and good nursing. Many great names are connected with the London Hospital, which, perhaps more than any other, is identified in the public mind with the personalities entrusted with its management. Mr. E. W. Morris, House Governor, has been actively concerned with it for some twenty-five years, and yet little is known of him outside his hospital activities. On these so much individuality has been spent that it becomes a question how much is left over for leisure and private interests and those prejudices without which no human being is complete.

*[Elliott & Fry.*

MR. E. W. MORRIS, C.B.E.

Like the Chairman, Lord Knutsford, Mr. Morris is an open-air man. Meredith once wrote a poem on the pleasure and advantage of shedding "rascal sweat," the pathetic tribute of the man of letters to the value of open-air exercise. Mr. Morris, unlike the character in the parable, not only can beg, but is not ashamed of digging; and when it is too wet to do that, he finds his pleasure on rainy days in a greenhouse. He cultivates varieties, and a new rose or a rockery in full flower in the spring gives him almost as much pleasure as a legacy to the Hospital. He likes to spend his summer holidays fishing, and plays golf at week-ends, though it is his humour to declare that he has never been undefeated. He too is an admirable letter-writer, and something more, as our own columns have often borne witness. There is a point and play of fancy in his illustrations that make him one of the most lively writers on hospital topics that we have. His history of the London Hospital was not only a contribution to the subject but, unlike many hospital histories, an acquisition to letters. There must rarely have been found a stronger combination than that of Lord Knutsford and himself. Both can speak, and both are ready writers, whose personalities never seem to get in one another's way, with the result that the London is run with the zest of a family party.

## Figures and Tables

**Figure f1:**